# Cases of Poisoning by Colored Confectionery, with Remarks

**Published:** 1850-07

**Authors:** H. Letheby

**Affiliations:** Lecturer on Chemistry in the London Hospital


### PATHOLOGY AND PRACTICE OF MEDICINE

*Cases of Poisoning by Coloured Confectionery, with Remarks.* By H. Letheby, M. B., Lecturer on Chemistry in the London Hospital.—Hannah Martin, aged 4½ years, Jane Embden, aged 10 years, and Amelia Leir, also aged 10 years, were admitted into the London Hospital on Sunday, April 28th ult., suffering from violent sickness and great prostration of strength.

It appeared that they had bought some sugared ornaments and colored confectionery from a Jew in Petticoat-lane; and that soon after they had partaken of these sweetmeats, they became very sick; complained of a burning sensation in the mouth, fauces, and œsophagus, of great pain in the stomach and abdomen, and were seized with violent retching, which was attended, after a few hours, with profuse purging. When they were admitted into the hospital they were seriously ill, for the features looked pale and shrunk, the extremities were deathly cold, the pulse was, in each case, small and feeble, and the surface of the body, especially of the last named child, was covered with a clammy perspiration. Emetics of sulphate of zinc were instantly administered, and the vomited matters were saved for analysis. Antidotes of milk, white of egg, and demulcents, were also given in great abundance; and, after the sickness had subsided, they were permitted to sleep, from which state they awoke considerably revived.

The vomited matters were evaporated to dryness, and the solid residue, not amounting to two drachms in weight, yielded abundant evidence of the presence of arsenic, copper, lead, iron, and zinc,—all of which metals, excepting the last named, had, doubtless, been derived from the confectionary of which the children had partaken.

On making inquiry into the matter, we were informed that between thirty and forty children had been attacked in a similar way, and that they had all purchased sweetmeats from the Jew in question; but it does not appear that he was acquainted with the poisonous nature of his merchandize, for he had purchased it (so he stated) as the refuse stock of a large and very respectable firm in the city.

It is not generally known that the ornamental kinds of confectionery are frequently tinted with poisonous pigments; that the greens, for example, are commonly produced by means of arsenite of copper, (Scheele's green,) verdigris, or a mixture of chrome and prussian blue; the yellow, by chromate of lead; the red, by vermillion, (bisulphuret of mercury,) or oxide of iron; and the whites, by carbonate of lead, oxide, or carbonate of zinc, chalk, or sulphate of baryta; and that, frequently, the fine frosting which covers the commoner kinds of twelfth-cakes, and the hard white sugar of comfits, contain from 10 to 30 per cent. of plaster of Paris or of whiting.

I have been induced to record the preceding cases, not so much for the purpose of exhibiting the nature of the symptoms observed, as with the view of showing the necessity for some legislative interference in a matter of what may truly be termed wholesale poisoning; for, without such evidence before the mind, it would not be credited by the great bulk of the community, that many of the prettiest and daintiest looking confections of the dessert-table are like the choice luxury of the Queen-mother, but too often the source of danger to those who partake of them.

Within the last three years no less than seventy cases of poisoning have been traced to this source; and how many, may we ask, have escaped discovery? In the month of September, 1847, Mr. Hetley, who is the visiting surgeon of St. Marylebone Infirmary, reported in the Pharmaceutical Journal, that he was requested, on the 14th of that month, to go as quickly as he could to the relief of some persons who had been taken suddenly and dangerously ill. He found three adults and eight children severely affected with vomiting and retching; the angles of their mouth and linen being colored green by the ejections. On seeking into the cause of this, he was told that one of the children had bought two pennyworth of some colored confectionary ornament, of which they had all partaken. Some of the offending article, (a thin cake of sugar and Paris plaster, covered with a layer of bright green) was, however, found, and it at once made the case clear.

In commenting on the above, Dr. Guy states, that "an accident on a larger scale, but happily unattended by any fatal result, occurred in our own experience,—one of the patients having been brought to the King's College Hospital on the day after the accident. An ornamental green basket, after having been used at an evening party, was given to one of the attendants, who distributed the fragments among the inmates of a large workshop. Severe vomiting and purging was the result. On inquiry at several confectionaries, we ascertained that arsenite of copper is commonly used to give a green color to confectionary, and an analysis of a fragment of the basket confirmed this statement."—*Ranking's Abstract*, Vol. VII., p. 347.

At the very time that the preceding article was going through the press, an inquiry was being instituted at Northampton before the county coroner, Mr. Hicks, respecting the death of Mr. William Cowfield, who, with twenty others, was poisoned at a public dinner given in that town, on the 7th of June, 1848, when it appeared that deceased had partaken of a blanc-mange, the top of which was colored with emerald green, (arsenite of copper,) and of which he died.

In the month of February, 1849, Dr. W. Fergus published the case of three children, who were poisoned by eating the green-sugared ornaments from a twelfth-cake. (*Med. Gaz.*, p. 304.) And, in the month of June following, Professor Christison exhibited to the members of the Edinburgh Medico-Chirurgical Society, a green powder which he had purchased at a confectioner's in that city. It was a portion of the stock employed to color jellies, &c.; and, on examination, he found that it consisted of sugar mixed with verdigris and arsenite of copper. His attention was drawn to it by the severe illness of two maid-servants who had partaken of some jelly colored with it.—*Lond. Journal of Medicine*, Vol. 1., p. 702.

Two years since, Professor Louyet, of Brussels, wrote to inform and caution us concerning the fact, that bon-bons, colored with an unusual quantity of chromate of lead, were being manufactured largely in London, and exported thence to Belgium. The bon-bons in question consisted of a species of aromatized sugar, colored yellow throughout its mass, exhibiting the scent and flavor of lemon, and encrusted with a species of transparent red-currant shell. Very recently some cheap almond and caraway and comfits have been sold at the grocers' and confectioners' in many parts of London, which are colored yellow by means of this pigment, for I have detected as much as half a grain of chromate of lead in three of these comfits.

This dangerous practice of coloring sweetmeats, &c., with poisonous substances is, unhappily, not peculiar to the English; for very recently some cases have been reported by MM. Houze and Jaubert, in which four persons were seriously attacked after having partaken of some bon-bons which were colored with arsenite of copper. One of the patients (a child aged six years) died from the effects of the poison, after an illness of two days; and a second child was brought so near to the grave that she did not recover for two years after the accident. So, again, it is recorded by Chevalier, that at a breakfast given on a festive occasion by an eminent Parisian lawyer, a boar's head was decorated in a very artistic matter with masses of fat, which were colored of a lively red and green tint. One of the guests, who was well acquainted with chemistry, suspecting that the pigment might be poisonous, retained a portion of the fat for further examination, and he found that it contained about two per cent. of arsenite of copper.—*Jour. de Chirur. Med.*, Jan. 1847.

All these facts, and there are many others of a like character which relate to the trade of the pickle-merchant, are sufficient to show that, however difficult it may be for the Home Secretary to give a correct definition of a poison, or even a complete list of poisonous substances, it is high time that the Government should take some steps to protect the lives of the community from danger, by imposing a sufficient check upon the present unrestricted sale and use of these, and such as these, the commoner poisons.—*London Med. Times.*

